# Supplementary material for: Trajectory of the arterial-alveolar oxygen gradient in COPD for a decade
Source: PLoS One. 2025 Jan 29;20(1):e0318377. doi: 10.1371/journal.pone.0318377 (PMC11778628; doi:10.1371/journal.pone.0318377)
Supplement: S1 Checklist — (DOCX) [file pone.0318377.s001.docx]

STROBE Statement—checklist of items that should be included in reports of observational studies

|  | Item No. | Recommendation | Page  No. | Relevant text from manuscript |
| --- | --- | --- | --- | --- |
| **Title and abstract** | 1 | (*a*) Indicate the study’s design with a commonly used term in the title or the abstract | 1 | Prospective cohort study |
|  |  | (*b*) Provide in the abstract an informative and balanced summary of what was done and what was found | 1 | A transition of the annual change of A-aDO_2_ from a stable state to a deterioration phase can serve… |
| Introduction | | | |  |
| Background/rationale | 2 | Explain the scientific background and rationale for the investigation being reported | 2 | the prognostic implications of A-aDO_2_ in patients with COPD have been scarcely explored… |
| Objectives | 3 | State specific objectives, including any prespecified hypotheses | 2 | our primary objective was to… |
| Methods | | | |  |
| Study design | 4 | Present key elements of study design early in the paper | 3 | We collected data for stable COPD patients who… |
| Setting | 5 | Describe the setting, locations, and relevant dates, including periods of recruitment, exposure, follow-up, and data collection | 3 | We collected data for stable COPD patients who visited Kyoto University Hospital between January 2006 and November 2008… |
| Participants | 6 | (*a*) *Cohort study*—Give the eligibility criteria, and the sources and methods of selection of participants. Describe methods of follow-up  *Case-control study*—Give the eligibility criteria, and the sources and methods of case ascertainment and control selection. Give the rationale for the choice of cases and controls  *Cross-sectional study*—Give the eligibility criteria, and the sources and methods of selection of participants | 3 | These patients were in exacerbation-free periods… |
|  |  | (*b*) *Cohort study*—For matched studies, give matching criteria and number of exposed and unexposed  *Case-control study*—For matched studies, give matching criteria and the number of controls per case | N/A |  |
| Variables | 7 | Clearly define all outcomes, exposures, predictors, potential confounders, and effect modifiers. Give diagnostic criteria, if applicable | 4 | The patients underwent baseline assessments at the beginning of the study. These assessments included… |
| Data sources/ measurement | 8* | For each variable of interest, give sources of data and details of methods of assessment (measurement). Describe comparability of assessment methods if there is more than one group | 4 | For all ABG analyses, we collected samples after the patient had rested for 15 minutes… |
| Bias | 9 | Describe any efforts to address potential sources of bias | N/A |  |
| Study size | 10 | Explain how the study size was arrived at | N/A |  |

Continued on next page

| Quantitative variables | 11 | Explain how quantitative variables were handled in the analyses. If applicable, describe which groupings were chosen and why | 5 | The change in pulmonary function and arterial blood gas parameters over a year was calculated and expressed as ΔParameter (/year). |
| --- | --- | --- | --- | --- |
| Statistical methods | 12 | (*a*) Describe all statistical methods, including those used to control for confounding | 5 | Continuous variables are expressed as means±SD unless otherwise specified… |
|  |  | (*b*) Describe any methods used to examine subgroups and interactions | N/A |  |
|  |  | (*c*) Explain how missing data were addressed | N/A |  |
|  |  | (*d*) *Cohort study*—If applicable, explain how loss to follow-up was addressed  *Case-control study*—If applicable, explain how matching of cases and controls was addressed  *Cross-sectional study*—If applicable, describe analytical methods taking account of sampling strategy | N/A |  |
|  |  | (*e*) Describe any sensitivity analyses | N/A |  |
| Results | | | | |
| Participants | 13* | (a) Report numbers of individuals at each stage of study—eg numbers potentially eligible, examined for eligibility, confirmed eligible, included in the study, completing follow-up, and analysed | 5 | We initially registered 220 patients. Then, 170 patients who underwent baseline and 157 who underwent follow-up… |
|  |  | (b) Give reasons for non-participation at each stage | Figure 1 |  |
|  |  | (c) Consider use of a flow diagram | Figure 1 |  |
| Descriptive data | 14* | (a) Give characteristics of study participants (eg demographic, clinical, social) and information on exposures and potential confounders | 5 | We initially compared the baseline characteristics of two groups: the CRF group (n=21), which needed LTOT within 10 years... |
|  |  | (b) Indicate number of participants with missing data for each variable of interest | Figure 1 |  |
|  |  | (c) *Cohort study*—Summarise follow-up time (eg, average and total amount) | 5 | We initially compared the baseline characteristics of two groups: the CRF group (n=21), which needed LTOT within 10 years, and the non-CRF group… |
| Outcome data | 15* | *Cohort study*—Report numbers of outcome events or summary measures over time | 5 | We initially compared the baseline characteristics of two groups: the CRF group (n=21), which needed LTOT within 10 years, and the non-CRF group (n=136) |
|  |  | *Case-control study—*Report numbers in each exposure category, or summary measures of exposure | N/A |  |
|  |  | *Cross-sectional study—*Report numbers of outcome events or summary measures | N/A |  |
| Main results | 16 | (*a*) Give unadjusted estimates and, if applicable, confounder-adjusted estimates and their precision (eg, 95% confidence interval). Make clear which confounders were adjusted for and why they were included | 5 | Our findings revealed that compared to the non-CRF group, the CRF group had significantly higher MRC dyspnoea scale scores, lower levels of PaO_2_, increased A-aDO_2_, reduced FEV_1_, and lower DL_CO_ and K_CO_ values (Table 1). |
|  |  | (*b*) Report category boundaries when continuous variables were categorized | N/A |  |
|  |  | (*c*) If relevant, consider translating estimates of relative risk into absolute risk for a meaningful time period | N/A |  |

Continued on next page

| Other analyses | 17 | Report other analyses done—eg analyses of subgroups and interactions, and sensitivity analyses | 7 | Next, we compared changes in pulmonary function tests and ABG analyses… |
| --- | --- | --- | --- | --- |
| Discussion | | | | |
| Key results | 18 | Summarise key results with reference to study objectives | 11 | In this study, we investigated the clinical importance of ABG parameters… |
| Limitations | 19 | Discuss limitations of the study, taking into account sources of potential bias or imprecision. Discuss both direction and magnitude of any potential bias | 14 | This study has several limitations. First, it was conducted in a single institution… |
| Interpretation | 20 | Give a cautious overall interpretation of results considering objectives, limitations, multiplicity of analyses, results from similar studies, and other relevant evidence | 14 | we did not evaluate sleep-disordered breathing, nor did we assess nocturnal hypoventilation or nocturnal hypercapnia. The longitudinal trend of ABG parameter… |
| Generalisability | 21 | Discuss the generalisability (external validity) of the study results | 14 | While this is consistent with the characteristics of patients previously reported in Japan, the results of this study may not necessarily be generalizable to individuals with obesity. |
| Other information | |  | | |
| Funding | 22 | Give the source of funding and the role of the funders for the present study and, if applicable, for the original study on which the present article is based | N/A |  |

*Give information separately for cases and controls in case-control studies and, if applicable, for exposed and unexposed groups in cohort and cross-sectional studies.

**Note:** An Explanation and Elaboration article discusses each checklist item and gives methodological background and published examples of transparent reporting. The STROBE checklist is best used in conjunction with this article (freely available on the Web sites of PLoS Medicine at http://www.plosmedicine.org/, Annals of Internal Medicine at http://www.annals.org/, and Epidemiology at http://www.epidem.com/). Information on the STROBE Initiative is available at www.strobe-statement.org.
