# Supplementary material for: Trajectory of the arterial-alveolar oxygen gradient in COPD for a decade
Source: PLoS One. 2025 Jan 29;20(1):e0318377. doi: 10.1371/journal.pone.0318377 (PMC11778628; doi:10.1371/journal.pone.0318377)
Supplement: S1 File — (DOCX) [file pone.0318377.s002.docx]

**Supporting Information**

**Trajectory of the Arterial-Alveolar Oxygen Gradient in COPD for a decade**

Kazuma Nagata^1^, Susumu Sato^1,2^*, Kiyoshi Uemasu^1†^, Naoya Tanabe^1^, Atsuyasu Sato^1^, Shigeo Muro^3†^, Toyohiro Hirai^1^

^1^Department of Respiratory Medicine, Graduate School of Medicine, Kyoto University, Kyoto, Kyoto, Japan

^2^Department of Respiratory Care and Sleep Control Medicine, Graduate School of Medicine, Kyoto University, Kyoto, Kyoto, Japan

^3^Department of Respiratory Medicine, Nara Medical University, Kashihara, Nara, Japan.

† These authors contributed equally to the work.

***Corresponding Author**:

Susumu Sato, MD, PhD

Department of Respiratory Medicine, Graduate School of Medicine, Kyoto University

Department of Respiratory Care and Sleep Control Medicine, Graduate School of Medicine, Kyoto University

54 Kawahara-Cho, Shogoin, Sakyo-Ku, Kyoto, 606-8507, Japan.

Tel: +81-75-751-3852

Fax: +81-75-751-3854

Email: ssato@kuhp.kyoto-u.ac.jp

**S1 Fig. Kaplan-Meier curves for OS without CRF among four groups divided using a two-by-two matrix, determined by the median values of baseline A-aDO_2_ and its 1-year change.**

Patients were divided into four groups using a two-by-two matrix, determined by the cutoff values of baseline A-aDO_2_ (23.75 Torr) and its 1-year change (ΔA-aDO2, 0.86 Torr/year), which were chosen based on optimal sensitivity and specificity in a multivariate ROC curve analysis. The groups were categorized as: “high&increased” (n=40), “high&sustained” (n=41), “low&increased” (n=45), and “low&sustained” (n=31). Log-rank test revealed significant differences among the curves (*P*<0.001). The hazard ratio for the "high&increased" group compared to the "high&sustained" group was 3.03 [95% confidence interval: 1.07 to 8.63] (p=0.04).


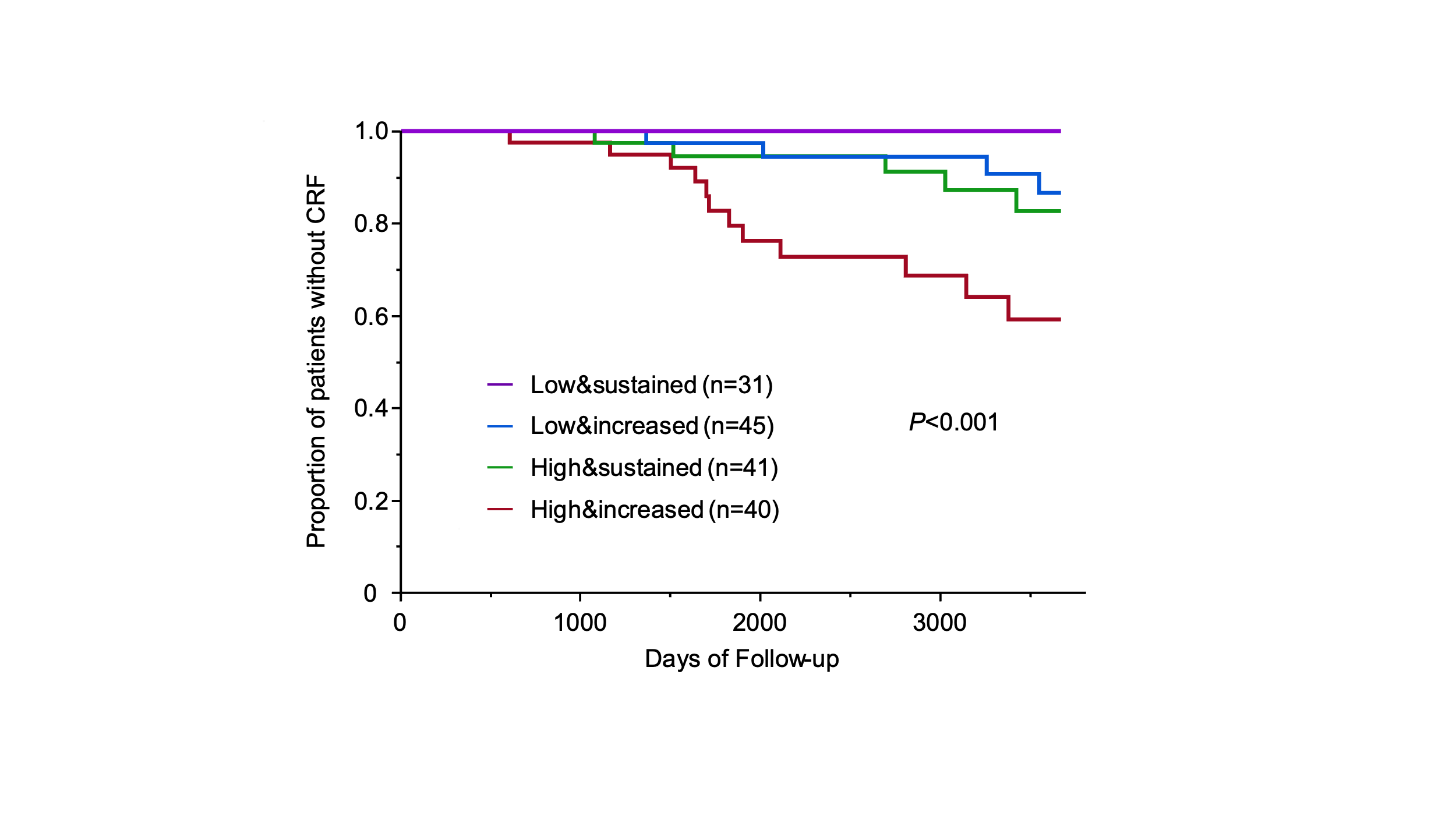


**S1 Table.** Number and proportion of patients with A-aDO_2_ of 30 Torr, 35 Torr, and 40 Torr or more each year tracked from the initiation of LTOT or the end of follow-up

|  | 9-10 | 8-9 | 7-8 | 6-7 | 5-6 | 4-5 | 3-4 | 2-3 | 1-2 | 0-1 |
| --- | --- | --- | --- | --- | --- | --- | --- | --- | --- | --- |
| **Non-CRF group** |  |  |  |  |  |  |  |  |  |  |
| **Total** | 36 | 34 | 32 | 27 | 28 | 29 | 28 | 29 | 29 | 36 |
| **≥ 30Torr** | 7 (19) | 6 (18) | 6 (19) | 7 (26) | 10 (36) | 6 (21) | 5 (18) | 8 (28) | 14 (48) | 7 (19) |
| **≥ 35Torr** | 2 (6) | 2 (6) | 3 (9) | 3 (11) | 3 (11) | 4 (14) | 2 (7) | 3 (10) | 3 (10) | 3 (8) |
| **≥ 40Torr** | 0 (0) | 1 (3) | 1 (3) | 1 (4) | 2 (7) | 2 (7) | 0 (0) | 1 (3) | 2 (7) | 0 (0) |
| **CRF group** |  |  |  |  |  |  |  |  |  |  |
| **Total** | 3 | 5 | 6 | 5 | 11 | 14 | 14 | 13 | 15 | 12 |
| **≥ 30Torr** | 2 (67) | 2 (40) | 4 (67) | 1 (20) | 4 (36) | 12 (86) | 11 (79) | 12 (92) | 13 (87) | 10 (83) |
| **≥ 35Torr** | 0 (0) | 1 (20) | 0 (0) | 1 (20) | 1 (9) | 9 (64) | 8 (57) | 10 (77) | 11 (73) | 9 (75) |
| **≥ 40Torr** | 0 (0) | 0 (0) | 0 (0) | 0 (0) | 1 (9) | 2 (14) | 4 (29) | 7 (54) | 5 (33) | 6 (50) |

A-aDO_2_, alveolar-arterial oxygen gradient; CRF, chronic respiratory failure; LTOT, long-term oxygen therapy.

In the period five to six years prior to initiation of LTOT or the end of follow-up, the proportions in the non-CRF group were 36%, 11%, and 7% for A-aDO_2_ of ≥30 Torr, ≥35 Torr and ≥40 Torr, respectively; the CRF group displayed similar ratios of 36%, 9%, and 9%, respectively, indicating little discernible difference between the groups at this stage. However, a notable disparity began to emerge from five years prior. More specifically, four to five years prior, the proportions in the non-CRF group were 21%, 14%, and 7% for A-aDO_2_ of ≥30 Torr, ≥35 Torr and ≥40 Torr, respectively, whereas in the CRF group, they notably escalated to 86%, 64%, and 14%, respectively. Subsequently, this discrepancy was either sustained at the same level or even further widened.

**S2 Table.** The slope of change and the average A-aDO_2_ for each period were retrospectively tracked from the initiation of LTOT or the end of follow-up.

|  | **CRF group** | **Non-CRF group** | ***P* value** |
| --- | --- | --- | --- |
| **Number of patients** |  |  |  |
| 5-10 years, Torr/year | 7 | 33 |  |
| 0-5 years, Torr/year | 13 | 33 |  |
| **Slope of change for each period** |  |  |  |
| 5-10 years, Torr/year | -0.745 | 0.687 | 0.12 |
| 0-5 years, Torr/year | 1.379 | -0.203 | 0.048 |
| **RMSE** |  |  |  |
| 5-10 years, Torr  0-5 years, Torr | 2.671  3.676 | 4.578  4.436 | 0.06  0.29 |
| **Average for each period** |  |  |  |
| 5-10 years, Torr | 28.81 | 22.95 | 0.058 |
| 0-5 years, Torr | 37.05 | 24.17 | <0.0001 |

ABG, arterial blood gas; A-aDO_2_, alveolar-arterial oxygen gradient; CRF, chronic respiratory failure; RMSE, root mean square error.

For the slope of change for each period, we calculated the annual rate of change in A-aDO_2_ for each period using a linear regression model. For the standard deviation of the residuals, we first calculated the residuals, which are the differences between the observed and predicted values, and then computed their standard deviation to measure the dispersion of these residuals. The average for each period was determined as the mean value of A-aDO_2_ during each period. We compared these values between the CRF group and the non-CRF group using a t test and calculated the p values.
